# Incompleteness and misclassification of maternal death recording: a systematic review and meta-analysis

**DOI:** 10.1186/s12884-023-06077-4

**Published:** 2023-11-15

**Authors:** Sahar M. A. Ahmed, Jenny A. Cresswell, Lale Say

**Affiliations:** Department of Sexual and Reproductive Health Research, World Health Organization, Geneva, Switzerland

**Keywords:** Maternal health, Measurement and monitoring, Maternal mortality, Mortality under-reporting

## Abstract

**Objective:**

To quantify the extent of incompleteness and misclassification of maternal and pregnancy related deaths, and to identify general and context-specific factors associated with incompleteness and/or misclassification of maternal death data.

**Methods:**

We conducted a systematic review of incompleteness and/or misclassification of maternal and pregnancy-related deaths. We conducted a narrative synthesis to identify methods used to capture and classify maternal deaths, as well as general and context specific factors affecting the completeness and misclassification of maternal death recording. We conducted a meta-analysis of proportions to obtain estimates of incompleteness and misclassification of maternal death recording, overall and disaggregated by income and surveillance system types.

**Findings:**

Of 2872 title-abstracts identified, 29 were eligible for inclusions in the qualitative synthesis, and 20 in the meta-analysis. Included studies relied principally on record linkage and review for identifying deaths, and on review of medical records and verbal autopsies to correctly classify cause of death. Deaths to women towards the extremes of the reproductive age range, those not classified by a medical examiner or a coroner, and those from minority ethnic groups in their setting were more likely misclassified or unrecorded. In the meta-analysis, we found maternal death recording to be incomplete by 34% (95% CI: 28–48), with 60% sensitivity (95% CI: 31–81.). Overall, we found maternal mortality was under-estimated by 39% (95% CI: 30–48) due to incompleteness and/or misclassification. Reporting of deaths away from the intrapartum, due to indirect causes or occurring at home were less complete than their counterparts. There was substantial between and within group variability across most results.

**Conclusion:**

Maternal deaths were under-estimated in almost all contexts, but the extent varied across settings. Countries should aim towards establishing Civil Registration and Vital Statistics systems where they are not instituted. Efforts to improve the completeness and accuracy of maternal cause of death recording, such as Confidential Enquiries into Maternal Deaths, are needed even where CRVS is considered to be well-functioning.

**Supplementary Information:**

The online version contains supplementary material available at 10.1186/s12884-023-06077-4.

## Introduction

Maternal mortality is an important measure of women’s health during the reproductive ages. The United Nations Maternal Mortality Estimation Interagency Group (UN MMEIG) is one of the entities that produce global maternal mortality estimates [20, 46]. Their most recent estimates found that, in 2020, 223 women died due to maternal causes per 100,000 live births, with the majority of these deaths being from preventable causes [46]. In recognition of its importance as a global indicator of women’s health and development, maternal mortality is the focus of Sustainable Development target 3.1: to reduce the global maternal mortality ratio to less than 70 deaths per 100,000 live births by 2030, with no country having a maternal mortality ratio (MMR) higher than 140.

Investing and prioritising interventions to reduce maternal mortality requires accurate and timely data on the levels and trends of maternal mortality. Interventions can furthermore be tailored to sub-national level, if the data is of adequate quality. The need for accurate measurement of maternal mortality has been emphasised by the WHO in the *Strategies toward Ending Preventable Maternal Mortality”* (EPMM) report published in 2015. The report highlighted a need to improve measurement systems and data quality and to ensure that all maternal deaths are counted [40]. The 2021 EPMM Strategy published on April 2021 further stressed WHO’s commitment to this recommendation [43].

Civil Registration and Vital Statistics systems (CRVS) are the preferred source of data for producing comparable maternal mortality statistics. Many low- and middle-income countries (LMICs) do not have complete and accurate CRVS systems, and instead rely on other data sources, such as, facility-based systems, district management information systems, and Health and Demographic surveillance systems. These systems often do not have adequate coverage, and rarely provide nationally representative estimates, as a result, LMICS often count on ad hoc population-based surveys, though these tend to be sporadic and untimely.

Even in countries with a well-functioning CRVS, mortality data are often incomplete. Estimation errors are common across different adult mortality indicators [28], but there can be additional challenges in the measurement of maternal mortality. These challenges include the additional information required to classify a death as maternal, such as accurate assignment of cause of death, pregnancy status and/or timing of the death relative to pregnancy.

Errors in maternal mortality estimation can be conceptually and operationally categorised into two types, 1) incompleteness (also known as missingness or under-reporting), defined as whether the death is registered into a designated data collection system (often a CRVS) or an alternative routine data reporting system. 2) misclassification refers to whether the cause of death is accurately documented, which affects whether the death is considered maternal or non-maternal, and is expressed as sensitivity and specificity of the surveillance system classification of cause of death [45]. Reducing incompleteness requires interventions aimed at establishing and/or improving death and cause of death registration systems, while reducing misclassification requires interventions aimed at improving the accuracy of cause of death classification, such as the use of the International Classification of Disease (ICD) for certifying and coding the cause of death [42].

Improving the performance of registration systems requires continuous monitoring of the extent of incompleteness and/or misclassification of maternal and pregnancy related deaths. Furthermore, understanding which subset(s) of maternal deaths are more likely to be incomplete or misclassified can provide insights for researchers and stakeholders in implementing targeted interventions or studies aimed at identifying and/or classifying maternal deaths.

Currently, the MMEIG estimates sensitivity and specificity of maternal death recording using a Bayesian hierarchical modelling approach. Due to the requirement of national representativeness for inclusion of studies, they do not estimate under-reporting and misclassification in non-CRVS surveillance systems, for example in facility based or in health and demographic surveillance systems, hence, their estimates may not generalise to non-CRVS systems. Such systems may still provide valuable information on maternal mortality, especially in countries with limited data. Furthermore, many countries are working towards improving the coverage of their surveillance systems, and in this review, we evaluate and compare the quality of maternal death recording and classification between several surveillance systems, in an attempt to provide insight into their quality for maternal mortality measurement.

The primary aim of this systematic review and meta-analysis is to quantify the extent of incompleteness and misclassification of maternal deaths, and describe the methods used to identify missing and misclassified maternal deaths. Additionally, we describe the socio-demographic and clinical characteristics of women whose deaths are more likely to be unreported or misclassified, and the key contextual factors associated with incompleteness and/or misclassification.

## Materials and methods

This study is a narrative review and a meta-analysis reporting on a sub-set of data from a larger systematic review of studies that report on the level of maternal and or pregnancy related deaths, and the completeness and/or misclassification of these deaths. The review was conducted as part of the UN MMEIG estimation process for the 2022 round of global MMR estimates [46].

The main outcomes for the meta-analysis were i) incompleteness, and ii) misclassification of maternal or pregnancy related deaths within a routine surveillance system (specificity and sensitivity). We used the ICD-11 definitions of maternal and pregnancy related deaths, and of direct and indirect obstetric deaths [44].

In the narrative review, we aimed to synthesise information on the methods used by the identified studies to obtain the true number of maternal deaths, and the context specific challenges they identified for measuring maternal mortality.

### Definition of key terms and measures

Sensitivity refers to the proportion of correctly classified maternal deaths out of all true maternal deaths. Specificity refers to the proportion of correctly classified non-maternal deaths out of all true non-maternal deaths. False negative referred to the maternal deaths that are incorrectly recorded as non-maternal. incompleteness refers to the proportion of the true maternal deaths identified in the population that were not previously recorded in the routine surveillance system. The proportion under-estimated was defined as the proportion of deaths not reported from the total maternal deaths, whether from incompleteness, false negatives, or both.

The above metrics likely differ according to the system being compared to, and we therefore stratified our analysis based on the comparison used. These categories were CRVS; national Health Management Information System (if the system described was instituted nationally, but was facility based); facility-based. (if it only includes deaths from one or several facilities but not all facilities in the country); Health and Demographic Surveillance system (if it was instituted in a given area (not national) and dedicated for ongoing demographic surveillance at both facility and community levels).

### Eligibility criteria

For a study to be included in the narrative review it had to satisfy the following criteria: either 1) provide empirical data on the completeness and/or misclassification of maternal and/or pregnancy related deaths or 2) conduct an investigation to obtain the true number of maternal and/or pregnancy related deaths by triangulating data sources, and the results of the investigation could be compared against a CRVS observation obtained from the WHO Mortality Database [39]. 3) all included studies must report the definition used to classify the deaths (maternal, pregnancy-related).

Studies were included in the quantitative analysis if they provided the number of missed or misclassified maternal or pregnancy related deaths in a surveillance system that is routinely in place in the area or facility covered by the study. We excluded from the meta-analysis studies that only provided a percentage of under-estimation from which we could not obtain the number of missed or misclassified deaths.

### Search strategy

We searched five bibliographic databases: Medline (OVID), EMBASE (OVID), EBSCO, Global Index Medicus, and Web of Science – Russian index (Russian-script). The searches covered publications indexed between September 2016 – when the searches for the 2019 Update were run, the results of this have been previously reported [31] – and March 2021. In addition to bibliographic databases, we searched National Statistics and Health Ministries’ official websites of the 194 UN Member States for any specialised studies that could satisfy the inclusion criteria described above. Finally, we screened the references of the studies that were included from the bibliographic search for additional sources. We used search terms relating to maternal and pregnancy related mortality, and under-reporting, incomplete recording or data verification related terms developed by Gülmezoglu et al, 2004 [17]. We only excluded articles published in Chinese, for lack of translators. The search terms are listed in Supplementary information 1.

### Screening & data extraction

The identified citations were screened independently by two reviewers (SA, FA). The review process was completed in two stages. In the first stage, duplicate studies (*n* = 463) were removed, and title and abstracts of the studies were screened based on the inclusion criteria described above.

In the second stage, the full text for the identified studies was obtained and reviewed, and their usability was further examined based on the above criteria by two reviewers. A third reviewer was consulted to adjudicate on discordant opinions. The screening was managed using DistillerSR application [14].

For studies passing this stage, two reviewers extracted information on the study site and date, study design, coverage, method of identifying and ascertaining cause of death, the comparator, extent of incompleteness, specificity and/or sensitivity of death reporting, characteristics of misclassified and/or missed (incomplete) deaths and any contextual moderators influencing the registration of maternal/pregnancy related deaths.

For studies that did not compare directly to a registration system (eligibility criteria 2), two reviewers extracted the number of maternal deaths from the WHO mortality database for the year the study is reporting on.

In the case of non-English manuscripts, the extractors used a translation software, and a native speaker was sought to validate that the extraction was accurate.

### Study risk of bias assessment

Risk of bias was assessed using a scale designed by the authors of this study, which evaluates the ability of the study to capture maternal deaths from four aspects. The first is whether the study methodology will enable it to identify specific deaths that have been reported in the literature to be prone to missingness or misclassification. These include early pregnancy deaths, indirect obstetric deaths (deaths due to a disease (other than HIV) aggravated by the effects of pregnancy and deaths occurring at home. For a study to identify these deaths, it should a) examine all deaths to women of reproductive age, including those occurring at home and in non-obstetric departments; and b) conduct its own review of cause of death using available data sources. The second aspect was the method of determining the cause of death. We considered a verbal autopsy or a medical record review to be inferior to a death certificate in a country with a complete CRVS – as defined in the MMEIG MMR estimation methodology [41] – and superior to a death certificate from a country with no or low completeness CRVS system. We considered a verbal autopsy equal to a medical review since it was difficult to determine the level of completeness of the records reviewed. A medical examiner or forensic report, or a combination of two or more of the above methods were considered the most robust.

Third was the percentage of records or deaths that were not reviewed or that did not have enough information to ascertain the cause of death.

The fourth and final aspect was the population coverage. We scored studies with nationally representative samples higher than those without, and studies that were population based, or facility based in a context where more than 95% of deliveries are attended by a skilled person were both scored higher than facility-based studies where less than 95% of were attended by a skilled birth attendant [39].

Studies scoring 1–2 were categorized as high risk of bias, 3–5 medium, and 6–8 low. Studies that scored high in the risk of bias were excluded from sub-group meta-analysis, except when we disaggregated by study risk of bias category. The scoring form is presented in Supplementary information 2.

This scale was chosen to evaluate criteria that are specific to maternal mortality identification and classification that wouldn’t be captured using standard risk of bias criteria.

### Synthesis methods

Qualitative narrative synthesis was done for all studies included in this review. We extracted information about the methodology used in the studies, the factors associated with incompleteness or misclassification and any context-specific challenges that may have affected the reporting of maternal or pregnancy related deaths (as defined in ICD-11 [44].

A meta-analysis of proportions was conducted for studies reporting 1) Incompleteness (the prevalence of under-reporting of maternal and/or pregnancy related deaths in a surveillance system operating in the study area), and for 2) the sensitivity of maternal cause of death registration in a surveillance system. Studies reporting on more than one study location where they provided numbers of total and missed/misclassified deaths for each location were treated as separate observations. We stratified incompleteness by cause of maternal death, place of death and by time of death relative to delivery due to data availability.

We finally calculated the pooled percentage by which maternal deaths were under-estimated either from incomplete recording, misclassification or both. The overall under-estimation was further stratified by the income level of the country and the surveillance system investigated.

We conducted a meta-analysis for the sensitivity, but not for the specificity due to the very small number of studies reporting it.

Statistical analysis was conducted using R studio, version 4.2.0. The variance of the proportions was used to weight estimates from each study and produce pooled estimates. The proportions from each study were transformed using a Freeman-Tukey type arcsine square-root transformation, and the DerSimonian-Laird random effects method was used to combine study estimates. Estimates were stratified based on the country income level, type of surveillance system, and the quality score of the study. We did not undertake sensitivity assessment for this model.

For studies that reported on incompleteness by the cause of maternal death (direct vs indirect), timing of death relative to pregnancy, and the place of death (home vs facility) we used the same methods above to pool the estimated incompleteness across the studies for each category, where possible.

To evaluate the consistency of the meta-analysis results we report H^2 and I^2. H^2 is a measure of heterogeneity in a meta-analysis, representing the ratio of observed variance to expected variance. A value greater than 1 suggests heterogeneity. I^2 quantifies the proportion of observed variance that can be attributed to differences between studies, rather than sampling error. An I^2 of 0% indicates no heterogeneity, 25% is low, 50% is moderate, and 75% or higher is high heterogeneity.

## Results

### Search results

**Fig. 1:**
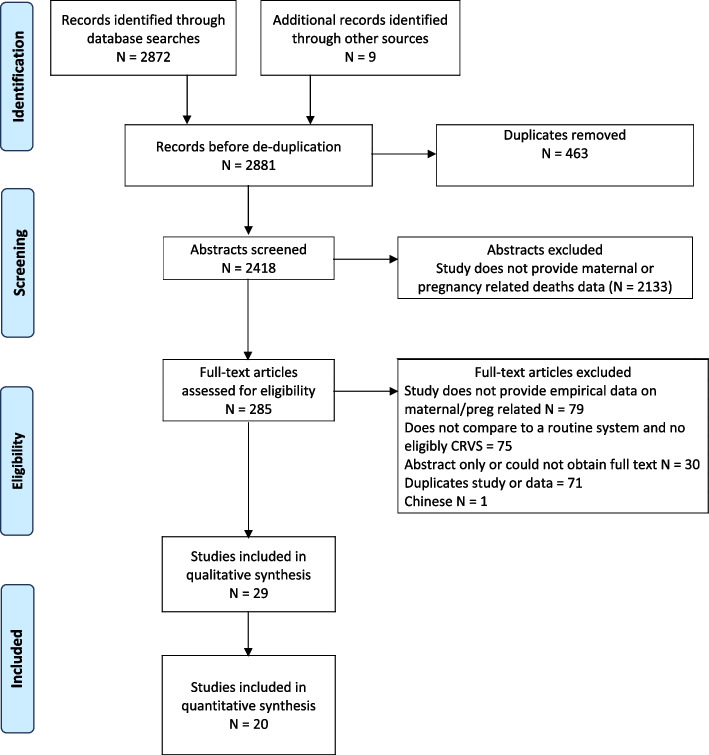
PRISMA flow chart summarising the search strategy

The results of our search strategy are presented in Fig. 1. In brief, 2872 records were identified through the searches of the bibliographic databases, of which 463 were duplicates. Six additional articles were identified from reviewing the reference lists of the eligible studies, and three more were obtained from searching government websites. After title-abstract screening, 285 sources were assessed for full-text screening and 29 were identified as eligible for inclusion. Of the 29 studies, 21 reported on the number of under-reported (incomplete) or misclassified deaths, and the total number of maternal deaths identified, thus were suitable for a meta-analysis, but one study [30] was excluded because their deaths were included in the aggregate of another included study [26], making the final number of studies included in the meta-analysis (*n* = 20). Two studies [10, 29] were excluded from subgroup analyses as they were considered to have high risk of bias.

### Study characteristics

The characteristics of included studies are summarised in Table 1. All the studies had a cross-sectional design, apart from one that was a nested case control study. The latter allowed for comparable data to be extracted and thus included in meta-analysis. More than two thirds of the studies were subnational (*n* = 19), and six studies were facility-based. More than half of the studies were considered to be of low risk of bias (*n* = 15), eight were medium and four were high. Most prevalent issues were the high percentage (> 10%) – or non-reporting – of deaths for which a cause of death could not be established. Furthermore, only seven studies had complete coverage (national and investigates all deaths to women of the reproductive age). Two manuscripts were in Spanish, and the remaining 27 were retrieved in English.

**Table 1:** General study characteristics

No	Study author & year of publication	Country	Income	Data collection period	Coverage	Study design	Comparator	Method of identifying maternal deaths	Study risk of bias	Meta analysis^e^
1	(AIHWs, 2020) [4]	Australia	High	2018	National – population based	Cross-sectional	CRVS	CEMD^a^	Low	A, F
2	(Berdzuli et al., 2020) [7]	Georgia	High	2012	National – population based	Cross-sectional	CRVS	Triangulation of data from different sources	Low	A, E, F
3	(Baeva et al., 2018) [6]	USA	High	2012	Subnational – population based	Cross-sectional	CRVS	Review of death records and data linkage	Medium	NA
4	(Boutin et al., 2021) [9]	Canada	High	2013—2017	Subnational – facility based	Cross-sectional	FBSS	Review of electronic medical records	Medium	NA
5	(Catalano et al., 2021) [11]	USA	High	2016	Subnational – population based	Cross-sectional	Pregnancy checkbox on death certificates	Data linkages (birth and foetal death registers)	Medium	NA
6	(Davis et al., 2017) [12]	USA	High	1987—2012	National – facility based	Cross-sectional	MMR in 1978 to 2008 (before introduction of pregnancy checkbox)	Review of national statistics records of mortality and natality files	High	NA
7	(Deneux-Tharaux et al., 2005) [13]	USA, Finland and France	High	1999—2000	Subnational – population based	Cross-sectional	CRVS	Data linkage (birth and foetal death registers)	Low	F
8	(Donati et al., 2018) [15]	Italy	High	2006—2012	Subnational—facility based	Cross-sectional	CRVS	Data linkages (pregnancy or pregnancy-related hospitalizations records)	Medium	NA
9	(Horon, 2005) [19]	USA	High	1993—2001	Subnational – population based	Nested case–control	CRVS	Data linkages (birth and foetal death registers)	Low	A, D, F
10	(Laura et al., 2020) [24]	Switzerland	High	2005—2014	Subnational – population based	Cross-sectional	CRVS	Data linkages (birth and foetal death registers)	Low	A, B, F
11	(MBRRACE-UK, 2020) [26]	United Kingdom	High	2016—2018	National – population based	Cross-sectional	CRVS	CEMD	Low	A, F
12	(Sesmero et al., 2016) [34]	Spain	High	2012	Subnational—facility based	Cross-sectional	FBSS	Questionnaire filled by heads of hospitals asking about maternal deaths information	Medium	NA
13	(O’Hare et al., 2020) [30]	Ireland	High	2016—2018	National – population based	Cross-sectional	CRVS	CEMD	Low	NA
14	(Vangen et al., 2017) [37]	Denmark, Norway, Finland, Iceland, Sweden	High	2005—2013	National – population based	Cross-sectional	CRVS	Triangulating data from different sources	Low	A, F
15	(Abalos et al., 2019) ^d^ [1]	Argentina	Upper-Middle	2014	National – facility based	Cross-sectional	CRVS	Review of clinical records	Medium	A, B, E, F
16	(Bess Constantén et al., 2018) ^d^ [8]	Cuba	Upper-Middle	2013	national – population-based	Cross-sectional	CRVS	Review of clinical records	Low	A, B, F
17	(Kodan et al., 2017) [21]	Suriname	Upper-Middle	2010–2014	National – population based	Cross-sectional	CRVS	RAMOS^b^	Low	A, E, F
18	(Lin et al., 2019a) [25]	China, Taiwan Province of China	Upper-Middle	2010—2017	Subnational – population based	Cross-sectional	Pregnancy checkbox on death certificates	Review of pregnancy checkbox field on death certificate	Medium	NA
19	(Wu et al., 2015) [47]	China, Taiwan Province of China	Upper-Middle	2000—2009	Subnational – population based	Cross-sectional	CRVS	Data linkage of CRVS with insurance claims data	Medium	A, E, F
20	(Abouchadi et al., 2018) [2]	Morocco	Lower-Middle	Jan 2013 – Sept 2014	Subnational – population based	Cross-sectional	CRVS	RAMOS	Low	A, B, C, D, F
21	(Anwar et al., 2018) [5]	Pakistan	Lower-Middle	Jun 2015 – May 2016	Subnational – population based	Cross-sectional	MMR from the 1.5 years before the study	follow up of pregnant women until 42 days post termination of pregnancy	High	NA
22	(Boyd et al., 2017) [10]	Haiti	Low	2014—2015	Subnational – facility based	Cross-sectional	FBSS	Review of clinical records and dossiers	High	A, F
23	(Garces et al., 2012) [16]	Philippines	Lower-Middle	2008	Subnational – population based	Cross-sectional	CRVS	RAMOS	Low	A, B, F
24	(Kodio et al., 2002) [22]	Senegal	Lower-Middle	1984—1995	Subnational – population based	Cross-sectional	HDSS	HDSS	Low	A, F
25	(Mswia et al., 2003) [27]	Tanzania	Lower-Middle	July 1993 – Dec 1999	Subnational – population based	Cross-sectional	HDSS	An active reporting system based on a network of respected individuals within each community	Medium	A, F
26	(Mwaniki et al., 2020) [29]	Kenya	Lower-Middle	Jan 2015 – June 2018	Subnational – facility based	Cross-sectional	FBSS	MDSR^c^	High	A, F
27	(Qomariyah et al., 2020) [32]	Indonesia	Lower-Middle	2016	Subnational – population based	Cross-sectional	DHIS	review of routine HIS in addition to asking village informants	Low	A, B, C, D, F
28	(Songane & Bergström, 2002) [35]	Mozambique	Lower-Middle	Aug 1996 – July 1997	Subnational – population based	Cross-sectional	DHIS	Triangulation from different data sources	Low	A, F
29	(Zakariah et al., 2009) [48]	Ghana	Lower-Middle	2002	Subnational – population based	Cross-sectional	DHIS	RAMOS	Low	A, C, F

^a^ *CEMD* Confidential Enquiry into Maternal Deaths

^b^ *RAMOS* Reproductive Age Mortality Survey

^c^MDSR: Maternal Death Surveillance and Response

^d^Manuscript in Spanish

^e^A = included in meta-analysis of incompleteness, B = included in meta-analysis of completeness by cause of death; C = included in meta-analysis of completeness by place of death; D = included in meta-analysis of completeness by time of death relative to delivery, E = included in sensitivity meta-analysis, F = included in analysis of under-estimation

Fourteen studies came from high income countries, five from upper-middle-income countries, and the remaining 10 were from low and lower-middle-income countries.

In general, studies from higher income countries more frequently had national coverage and lower risk of bias and tended to compare against a CRVS. Nine out of the 19 high and upper-middle-income studies had national coverage, they mostly investigated a CRVS (14/19) and only one had a high risk of bias score. In contrast, the ten Low and Lower Middle-Income Country studies were all subnational (*n* = 10), three were of high bias score, and only one made a comparison against a CRVS system.

Of the 20 studies eligible for quantitative synthesis, 15 were of low risk of bias, three were medium and two were high risk (supplementary information 3). One study reported on pregnancy related deaths, and the rest reported on maternal deaths. The two high risk of bias studies – which included the one reporting pregnancy related deaths – were only included when calculating the total under-estimation prevalence, and when we disaggregated under-estimation by risk of bias score.

### Methods used to assess the validity of maternal/pregnancy related death data

Thirteen studies reviewed medical/clinical records, sometimes in addition to other sources (forensic reports, death certificates, and criminal reports) to identify maternal deaths. Twelve studies linked or triangulated data from multiple sources to identify incompletely recorded or misclassified deaths. In most cases, the sources used were death certificates, birth and fetal death registers, and/or hospital records often using a unique identification number.

Three studies used the capture-mark-recapture methodology, namely, Haiti, Indonesia and the Philippines [10, 16, 32]. This method is used in public health to determine the size of populations that are difficult to identify [23], and requires four critical assumptions to be met: 1) a population is fixed,2) individuals from the two sources can be linked,3) capture in the second sample is independent of capture in the first sample; and 4) the probability of capture does not differ between individuals. The study from Indonesia used the District Health System, and interviews with village informants and health volunteers to capture all maternal deaths [32]. In Haiti, the two sources were a register data capture form and the dossier data capture form [10]. In the Philippines study, they used vital registration and the second source was a Reproductive Age Mortality Survey [16].

The three studies concluded that no single data source was able to capture all deaths. In Indonesia and the Philippines, 49% and 44% of deaths were missed by one of the two sources respectively. In Haiti, where both sources were facility-based, only about a quarter of deaths were captured by each source. The study from Haiti however could not guarantee the first third, and fourth assumptions of the capture-mark-recapture method were met in their study.

A smaller subset of studies (*n* = 4) used active surveillance and notification to identify maternal deaths, and only one study followed all pregnant women to identify any deaths.

In total, 1*9* studies conducted their own independent review of cause of death to quantify misclassification. The studies mostly reviewed cause of death using either a verbal autopsy, review of medical records, or a combination of both (Table 2).

**Table 2:** List of studies that conducted a review of maternal cause of death, with the review methods, and result of the review

Study	Country	Review method	Maternal deaths originally recorded	Result of the review^a^
Abouchadi et al. 2018 [2]	Morocco	Verbal autopsy and review of medical records and death certificate	32	17 missed 23 F-
AIHW, 2020 [4]	Australia	Review of medical records and death certificates	13	2/15 missed
Boutin et al.2020 [9]	Canada	Review of medical records	No comparison	No comparison
Boyd et al., 2017 [10]	Haiti	Review of medical records	86	25 missed
Constanten et al., 2018 [8]	Cuba	Review of medical records, verbal autopsy and forms filled by attending physician	49	1 missed
Deneux-Tharaux, 2005 [13]	USA	Review of medical records, medical examination records and death certificates	3	3 missed
Donati et al., 2018 [15]	Italy	Expert review of medical records	150	58 missed
Graces et al., 2012 [16]	Philippine	Verbal autopsy	14	46 missed
Kodan et al., 2017 [21]	Suriname	Review of medical records	48	17 missed 31 F-
Kodio et al., 2002 [22]	Senegal	Verbal autopsy	64	20 missed 2 F +
Laura et al., 2020 [24]	Switzerland	Review of medical records, and other available documentations	4	1 missed
Berdzuli et al, 2020 [7]	Georgia	Verbal autopsy	12	9 F-
MBRRACE-UK, 2020 [26]	UK	Review of medical records and other available documentations	117	111 missed
Mswia, et al., 2003 [27]	Tanzania	Verbal autopsy	43	64 missed
Mwaniki, Edwards & Kizito, 2020 [29]	Kenya	Review of medical records and other documentation	42	1 missed
Qomariyah et al., 2020 [32]	Indonesia	Verbal autopsy	95	74 missed
Songane & Bergström, 2002 [35]	Mozambique	Medical records and other documentations, and verbal autopsy	6	34/40 missed
Vangen et al., 2017 [37]	(Nordic countries)	Review of medical records and other documentation	60	68/128 missed
Wu et al., 2015 [47]	China, Taiwan Province of China	Review of medical records and deaths certificates	9	17/26 F-

^a^ *F-* false negative, *F* + false positive

### Incompleteness of maternal death recording

There was a wide range in the extent of incompleteness, from 0 to 85% across 16 studies, and a pooled proportion of 34% (95% CI: 28–48). We found high between-study heterogeneity across all pooled estimates of incompleteness (I^^2^ = 91.2%; *P* < 0.001) (Table 3).

**Table 3:** Random-effects meta-analysis of pooled prevalence of incompleteness and sensitivity of maternal deaths recording stratified by individual covariates (excluding two studies for high risk of bias score)

Category	Number of studies	Pooled estimate % (95% CI)	Variation due to study heterogeneity
Overall sensitivity	4	61% (37–82)	I^2 = 92.7 H^2 = 13.7 (*P* < 0.001)
Total incompleteness	16	34% (28–48)	I^2 = 91.2% H^2 = 12.3 (*P* < 0.001)
*Incompleteness by cause of maternal death*			
Direct	6	22% (4 – 48)	I^2 = 96.1% H^2 = 25.3 (P = 0.006)
Indirect	6	42% (10–76)	I^2 = 87.9% H^2 = 8.3 (*P* < 0.001)
*Incompleteness by place of death*			
Home	3	75% (20–100)	I^2 = 96.4% H^2 = 27.6 (*P* < 0.001)
Facility	3	27% (6–58)	I^2 = 96.0% H^2 = 24.8 (*P* < 0.001)
*Incompleteness by time of death relative to pregnancy*			
During delivery or within 24 h	3	25 (16–33)	I^2 = 0% H^2 = 1 0.0 (P = 0.738)
During pregnancy	3	52 (39–64)	I^2 = 0.0 H^2 = 1 0.0 (P = 0.422)
More than 24 h post-partum	3	52 (40–63)	I^2 = 36.4% H^2 = 1.7 (P = 0.239)

Across the six studies that stratified by cause of death, incompleteness for indirect deaths was higher than for direct deaths (42% and 22% respectively), though confidence intervals were very wide and overlapped substantially (10 -76% and 4—48% respectively). There was evidence of high between study variability in both categories (I^2^: 96.1% & 87.9% respectively; *P* < 0.001).

Among three studies stratifying by place of death, incompleteness was higher for death that occurred at home: 75% versus 27% incompleteness, albeit with a notable overlap in the confidence intervals (95%CI 20 to 100 & 6 to 58 respectively). There was strong evidence of between study variability (I^2 = 96.4; & 96.0 respectively) (Table 3).

Deaths occurring either during pregnancy or after 24 h postpartum had a higher incompleteness (52%) compared to deaths occurring during delivery or within 24 h postpartum (25%), with some overlap in the confidence intervals. Notably, there was no evidence of between study heterogeneity in the three categories (Table 3).

Only one study stratified unregistered deaths by maternal age: this study found that deaths at the extremes of maternal age (less than 20 and above 40) were more frequently missed; half of all maternal deaths among adolescents and more than half of all maternal deaths among women aged 40 and above were under-reported, while 28% were missed in the 20–39 age group [19].

### Misclassification of maternal deaths

Sensitivity ranged from 10 to 86% across four studies, and the pooled estimate of sensitivity was 61% (95% CI 37–82) (Table 3). There was only one study reporting information about specificity and found it to be high (98.9%) [22].

Reported characteristics more prone to misclassification were the cause of maternal death being indirect, extremes of maternal age, the certifier being a physician rather than a coroner or medical examiner, and the deceased being a minority ethnic group (Lin et al., 2019b) [6, 11]. However, the number of deaths in these studies was too low to determine statistical significance.

Three studies (two from USA and one from China, Taiwan Province of China) looked at the impact of adding the pregnancy checkbox to the death certificate [12] [11, 26],they found it led to the identification of more maternal deaths and therefore an increase in the MMR (from 9 to 22 in the states it was implemented in in the US,from 55 to 82 in China, Taiwan Province of China, per 100,000 live births). However, they also found the checkbox led to an increase in the number of “false positives” and hence, may over-estimate maternal mortality if it is the sole reason for classifying a death as maternal.

### Overall under-estimation of maternal deaths

**Fig. 2:**
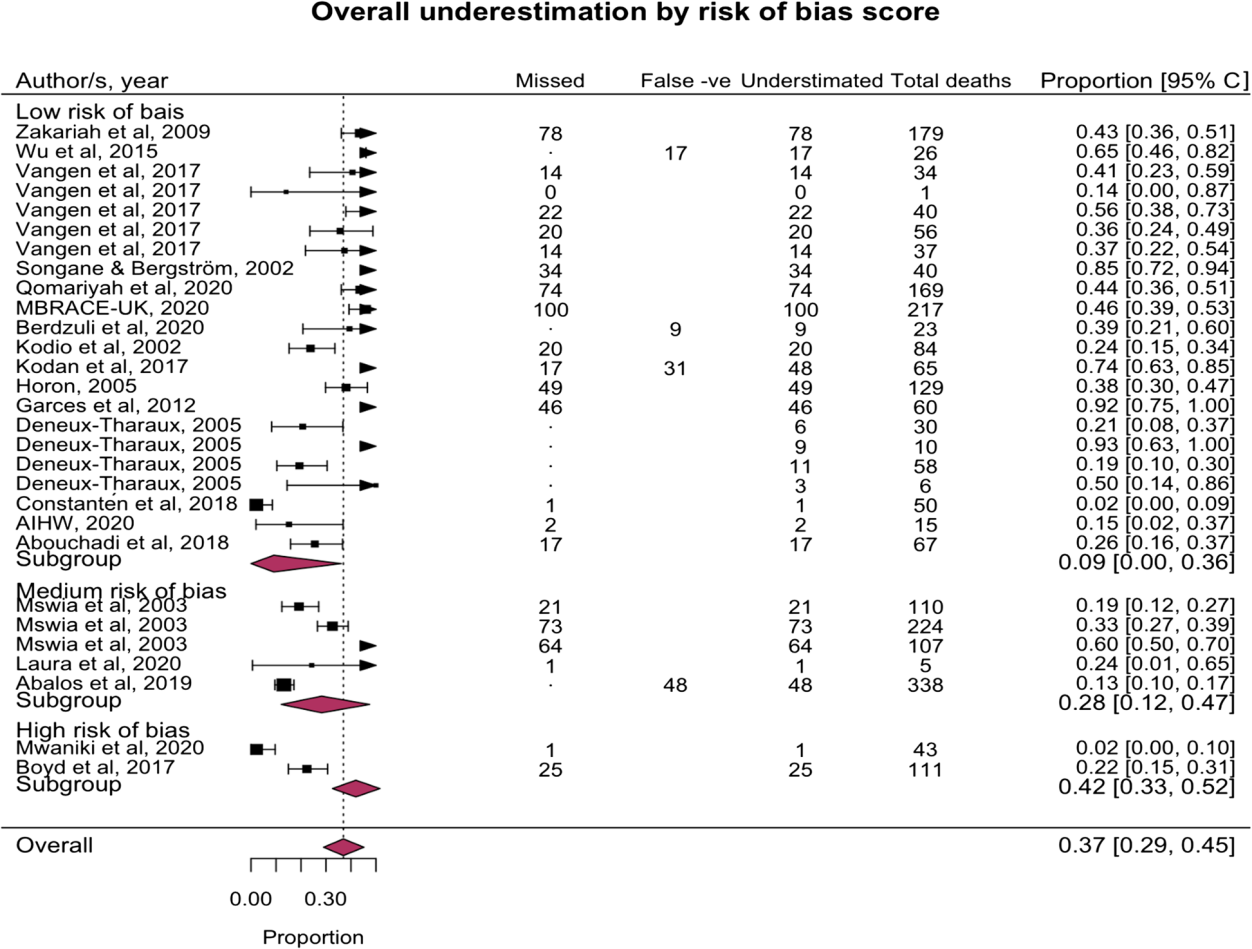
Forest plot of the pooled under-estimated proportion of maternal deaths (from incomplete reporting, misclassification (false negatives), or both) by study quality: random effects model with DL transformation. Caption: Fig. 2 shows the proportion of maternal deaths under-estimated for each study, the pooled proportion for each risk of bias sub-group, and the overall pooled proportion for all sub-groups. The lines opposite each study indicate the 95% confidence interval of the D-L transformed proportion. The box indicates the point estimate and the size of the black box indicates the “weight” of the study, or how much the study contributes to the sub-group and overall pooled proportion. A study with a bigger box has more influence. The triangles indicate that the confidence interval of the study is wider than the x -axis scale, and the direction of the tip of the triangle indicates in which direction the confidence interval is wider than the x-axis

Across 20 studies, underestimation ranged from 0% in Iceland to 85% in Mozambique, with a pooled proportion of 37%, due to incompleteness, misclassification (false negatives), or both. Heterogeneity between studies was high (I^2 = 93.3%; *P* < 0.001) (see Fig. 2). We found some evidence (*P* = 0.05) that studies with higher risk of bias score reported higher underestimation (9% 95%CI: 0—36) compared to medium and low risk of bias studies (28% 95%CI: 12—47 and 42% 95%CI: 33–52 respectively). When excluding the two studies with a high risk of bias score, pooled underestimation rose to 39% (95%CI: 30—48).

Under-estimation was higher in studies investigating District or Health Information Management Systems, compared to those investigating a CRVS or an HDSS (49%, 39% and 32% respectively), however there was no evidence of between group difference (*p* = 0.44), with a notable overlap in the 95% CI (Table 4). The under-estimation proportion was also higher among studies from low or lower middle-income settings (45%) compared to those from high/upper middle-income countries (36%), also with no evidence of between group heterogeneity (*p* = 0.36) (Table 4). Finally, under-estimation in studies where the mid-year of reporting is after 2010 was lower than those with mid-year between before 2000 and between 2000–2010, again with no evidence of heterogeneity between groups, and notable overlap in the confidence intervals.

**Table 4:** Pooled proportion of under-estimated maternal deaths, disaggregated by study covariates

Factor	Number of studies	Pooled proportion of under-estimation	Variation due to study heterogeneity
Total proportion of underestimated maternal deaths (exc high risk of bias studies)	18	39% (31–48)	I^2 = 93.1 H^2 = 14.3 *p* < 0.001
Type of system	Heterogeneity between groups p = 0.44		
CRVS	16	39% (27–51)	I^2 = 93.2 H^2 = 14.8 %; *p* < 0.001
Health and Demographic surveillance system	2	32% (16–50)	I^2 = 93.4 H^2 = 15.2 *P* < 0.001
District Health Information System or Health Management information system)	4	49% (42–75)	I^2 = 92.65 H^2 = = 13.6 *p* < 0.001
Country income level	Heterogeneity between income groups: p = 0.36		
High or upper middle-income country	16	36% (25–48)	I^2 = 92.2% H^2 = 12.8% (*P* < 0.001)
Low or lower-middle-income	9	45% (31–58)	I^2 = 94.1% H^2 = 16.8% (*P* < 0.001)
Mid-year of data collection	Heterogeneity between groups: p = 0.38		
Before 2000	4	42% (26–58)	I^2 = 93.1 H^2 = 14.5 (*P* = < 0.001)
2000–2010	6	47% (37–57)	I^2 = 76.7 H^2 = 4.3 (*P* = < 0.001)
After 2010	8	29% (14–48)	I^2 = 96.1 H^2 = 25.5 (*P* = < 0.001)

### Context specific challenges in classifying or registering maternal deaths

Broadly-speaking, there were three groups of challenges to recording maternal deaths. The first was lack of documentation and/or inadequate storage of medical records. One study from Haiti reported that out of 373 deaths to women of reproductive age, there was not sufficient information to determine cause of death for 56.3% of them, due to lack of documentation, or because medical records were damaged in storage [10]. In Switzerland, they could not determine cause of death for seven deaths (of 117 total) due to paucity of information available for reviewers [24].

Second was, challenges related to stigma, such as cultural beliefs about pregnancy and/or its termination. In Tanzania, induced abortion is illegal, and researchers identified this as a limitation to capturing resulting deaths [27].

Third, some studies identified issues arising from how the process of death notification/recording was organised. In, there were two separate electronic systems for recording deaths, and they were not sufficiently synchronised leading to one system not including any questions about pregnancy status while the second does [25]. Indonesia relies on a village midwife covering an area, urban or rural, for measuring maternal deaths, and with urban areas being more populated, the incompleteness was higher in urban areas compared to rural. Additionally, urban areas had more private clinics which may have led to midwives not being able to capture all deaths [32].

## Discussion

Our systematic review found substantial issues in the reporting of maternal deaths, with overall around a third of deaths not recorded in the studies examining different types of routine data systems. Sensitivity of maternal cause of death reporting was found to be 59%, and specificity from only one observation was 98%. These results align with the estimates of sensitivity and specificity produced by the Maternal Mortality Estimation Interagency Group’s Bayesian misclassification model (Sensitivity of 58% and Specificity of ~ 99%) [41]. The level of incompleteness and misclassification varied significantly between different contexts but was present across all settings. The combination of incompleteness and misclassification resulted in maternal deaths being underestimated by nearly 40%, though this is likely an under-estimate, since not all studies investigated both incompleteness and misclassification.

We found significant heterogeneity across most of the result, with the exception of incompleteness when disaggregated by cause of death (direct or indirect) and timing of death relative to delivery.

A higher proportion of maternal deaths were under-estimated in studies carried out in low and lower-middle-income countries (45%), compared to high and upper-middle- income countries (36%). A Health Management Information System appeared to miss more deaths (49%) than a CRVS or an HDSS (36% and 33% respectively). Additionally, studies with a low risk of bias reported less under-estimation than those with a high risk of bias score; these observations likely have common route causes.

We found the recording of indirect maternal deaths, deaths occurring at home and death further away from delivery (during pregnancy or after 24 h postpartum) to be less complete than their respective counterparts. The reasons for the latter two can be related to the prevalence of facility-based births in a country, coupled with the coverage of the surveillance systems to deaths which may occur outside of health facilities. Further, early pregnancy deaths maybe missed because the woman’s pregnancy status may not be known, or due to cultural barriers in countries where extramarital pregnancy is socially stigmatised. Deaths after 24 h postpartum will be more likely to happen at home or away from the obstetrics ward and thus missed.

Our finding of indirect maternal death reporting being less complete than for direct deaths requires attention, given the current trend of increasing maternal age and obesity globally, which may lead to an increase in the proportion of maternal deaths that are indirect. This trend forms the basis of the *obstetric transition*, in which countries either have, or are shifting from high to lower maternal mortality, and from a predominance of direct causes to indirect ones [36].

When the cause of death is not directly related to pregnancy, it can occur in non-obstetric departments/referral centres and likely outside the intrapartum period. Individuals classifying the cause of death, hence, may need to be prompted to check if any Women of Reproductive Age is pregnant or postpartum at the time of death via a pregnancy checkbox in the death certificate. The usefulness of this intervention was demonstrated in our review, where studies reporting on the validity of the pregnancy checkbox found that it did indeed lead to the identification of more maternal deaths.

The pregnancy checkbox is also present in the WHO’s International Form of Medical Certificate of Cause of Death, last updated in 2016, to guarantee the recording of minimum information required to code cause of death consistently across countries [44]. The WHO certificate has been adopted in some countries, but still not routinely used in others, and where it is used, it is sometimes not filled correctly or to completion [18]. Needless to say, the pregnancy check-box should not be the sole reason for classifying a death as maternal, to avoid over-estimation.

We found fewer studies reporting on misclassification compared to incompleteness, likely because identifying misclassified deaths requires additional information on the correct cause of death, and a wider sample frame. This was particularly true for specificity; studies tended to investigate deaths with a maternal cause of death or with evidence of pregnancy, allowing them to identify true positives and false positives, and some false negatives. They rarely however identified true negatives, as these would, in addition to correct cause of death, require investigating all deaths to women of the reproductive age, including those with no evidence of pregnancy. As a result, we could not evaluate over-estimation of maternal deaths. We argue, however, that in most contexts, under-estimation of maternal mortality is often more prevalent than over-estimation [3, 33], as demonstrated by the low sensitivity of maternal deaths compared to sensitivity both in this review and MMEIG Bayesean model [31].

Our review had a number of strengths. Firstly, we implemented a comprehensive search strategy, with broad search terms and no language restrictions to insure the identification of a good number of eligible studies. The literature was also supplemented by searching of government websites and reference lists, and also using CRVS maternal mortality estimates from the WHO mortality database to provide a comparator for studies which did not provide a comparison in their report. Secondly, the narrative synthesis we conducted enabled us to contextualise and better interpret the results obtained from the quantitative synthesis and meta-analysis. Thirdly, we were able to allow for more flexibility in estimating incompleteness and misclassifications in systems that would not be evaluated in the current MMEIG maternal mortality estimates, due to representativeness concerns. This allowed to us to compare the quality of reporting between different surveillance systems.

However, there were also some important limitations. First, the low number of studies reporting on incompleteness and more so on misclassification, especially in lower income settings. The search conducted was limited to studies indexed between 2016 – 2021, which may have reduced the number of potential studies. This was mitigated slightly by searching the reference lists of identified studies, allowing us to include older relevant sources. This limitation is further exacerbated by the low number of maternal deaths as an event, hindering our ability to make conclusive inferences on when and which deaths are incomplete or misclassified.

Secondly, we noted substantial heterogeneity in contexts and surveillance systems. These systems in general serve similar purposes, but how they are organised in a given country/context, their inadequacies, and challenges vary substantially. This is clear from the high estimates of heterogeneity (> 60%) throughout most of our results. This was also demonstrated in our synthesis of contextual challenges reported in the literature, where some studies identified issues specific to the system or country they validated. The heterogeneity means that our results cannot be generalised and must be interpreted with caution.

The methodologies employed in the included studies can provide useful indications on how to improve identification and classification of maternal deaths. One common technique was the linking of records from several sources to identify missed maternal deaths. The importance of this approach was highlighted in the three studies using the capture-mark-recapture methodology, where all three studies found not one single source was able to capture all deaths [16,33, 10] Another important element is the expert review of cause of death, which is an essential element of confidential inquiries into maternal deaths. The aim of confidential enquiries is, in addition to identifying an accurate cause of death, to highlight missed opportunities and to prevent the reoccurrence of similar deaths in the future [26]. Finally, we highlight the importance of investigating all deaths to WRA, and not only those with evidence of pregnancy.

We consider a CRVS to be superior to other surveillance systems investigated here, primarily due to its national-level coverage. Nonetheless, DHIS and HDSS systems can potentially provide valuable information on maternal mortality in LMIC, if data emerging from them is interpreted in the light of their limitations. HDSS data seem to be of relatively better quality than DHIS, however they are often sub-national, covering a relatively small area, and hence do not provide a national estimate of maternal mortality. HDSS data is a valuable resource that has been used to validate different mortality estimation methods in low- and middle-income settings, and can be used to inform and monitor the development and quality of routine sources, including a CRVS.

In conclusion, maternal mortality is substantially under-estimated in almost all contexts, but to varying degrees. Efforts to implement well-functioning CRVS systems are key to ensuring that all maternal deaths in a country are recorded and accurately classified. Where CRVS is instituted and functional, countries should continuously evaluate the completeness and accuracy of maternal deaths recording in the CRVS, perhaps through confidential enquiries. The WHO has produced guidance on improving the measurement of maternal mortality that is aimed at national level professionals [45]. This guidance provides valuable insights that address the measurement challenges identified in this review.

### Supplementary Information

**Additional file 1: Supplementary information 1.** Search terms used in the bibliographic search

**Additional file 2: Supplementary information 2.** Study risk of bias scoring form

**Additional file 3: Supplementary information 3.** Study risk of bias score for included studies

## Data Availability

Data used in the quantitative analysis is publicly available from reviewed studies manuscripts, and from the WHO mortality database.
