# Supplementary material for: Incompleteness and misclassification of maternal death recording: a systematic review and meta-analysis
Source: BMC Pregnancy Childbirth. 2023 Nov 15;23:794. doi: 10.1186/s12884-023-06077-4 (PMC10647144; doi:10.1186/s12884-023-06077-4)
Supplement: Supplementary file 1 — Additional file 1: Supplementary information 1. Search terms used in the bibliographic search [file 12884_2023_6077_MOESM1_ESM.docx]

# Supplementary information 1: Search terms used in the bibliographic search

#### English search terms:

| **No** | **Searches** |
| --- | --- |
| 1 | 'maternal mortality'/exp OR 'maternal mortality' OR 'maternal mortalities' |
| 2 | 'underreporting' OR 'under reporting' OR underreported OR 'under reported' OR 'data quality' OR 'official figures' OR 'record linkage' OR 'quality of information' OR 'officially reported' OR 'multiple sources' OR 'linkage' OR 'under registered' OR 'under registration' OR underregistered OR underregistration OR 'under registering' OR 'source of error' OR 'misclassification' OR 'misclassified' OR (errors AND ('registration'/exp OR registration)) OR 'late maternal mortality' OR 'confidential enquiries' OR 'confidential enquiry' |
| 3 | 'data collection method'/exp OR 'health survey'/exp AND (standard* OR method*) |
| 4 | #1 AND (#2 OR #3) |
| 5 | 'pregnancy'/exp OR 'pregnancy complication'/exp OR 'pregnancy disorder'/exp OR 'abortion'/exp AND ('death'/exp OR deaths OR 'mortality'/exp OR fatal OR fatalities OR deceased) |
| 6 | #2 AND #5 |
| 7 | #4 OR #6 |
| 8 | #4 OR #6 AND [1990-2016]/py |

| **No** | **Searches** |
| --- | --- |
| 9 | 'maternal mortality'/exp OR "maternal mortality" OR 'maternal mortalities' OR mortality OR "Hospital Mortality" OR 'Maternal Death'/exp OR Death OR "Death Certificates" OR Suicide OR Death OR deaths  OR Mortalities OR fatal OR fatalities OR "verbal autopsy" OR "verbal autopsies" OR "fatal" OR "survival rate" OR "survival rates" OR suicid* OR murder* OR homicide* |
| 10 | 'underreporting' OR 'under reporting' OR underreported OR 'under reported' OR 'data quality' OR 'official figures' OR 'quality of information' OR 'officially reported' OR 'under registered' OR 'under registration' OR underregistered OR underregistration OR 'under registering' OR 'source of error' OR 'misclassification' OR 'misclassified' OR (errors AND ('registration'/exp OR registration)) OR 'late maternal mortality' OR 'confidential enquiries' OR 'confidential enquiry' OR "missing data" OR "missed data" OR "missed figures" OR "inaccurate data" OR "inaccurate figures" OR "concealed numbers" OR "concealed figures" OR "hidden numbers" OR "hidden figures" OR "concealed data" OR "hidden data" |
| 11 | 'Pregnant Women'/exp OR 'Pregnancy Complications'/exp OR 'pregnancy'/exp OR 'Parity'/exp OR 'Maternal Health Services'/exp OR 'Mothers'/exp OR 'Maternal Welfare'/exp OR 'Maternal-Fetal Relations'/exp OR 'Maternal Exposure'/exp OR 'Maternal Behavior'/exp OR 'Gravidity'/exp OR 'abortion, legal'/exp OR 'Abortion, Criminal'/exp OR 'Abortion, Induced'/exp OR 'abortion, spontaneous'/exp OR 'Delivery, Obstetric'/exp OR "gestational diabetes":ti,ab OR eclampsia:ti,ab OR preeclampsia:ti,ab OR obstetric:ti,ab OR Gestation*:ti,ab OR Puerperal:ti,ab OR Puerperium:ti,ab OR Antepartum:ti,ab OR I3ntrapartum:ti,ab OR Postpartum:ti,ab OR "post partum":ti,ab OR Parturition:ti,ab OR perinatal:ti,ab OR Postnatal:ti,ab OR peri-natal:ti,ab OR "post natal":ti,ab OR "ante natal":ti,ab OR antenatal:ti,ab OR Miscarriage*:ti,ab,de,tn OR Maternity:ti,ab OR Maternal*:ti,ab OR mother*:ti,ab  OR Cesarean:ti,ab OR Caesarean:ti,ab OR c-section:ti,ab OR c-sections:ti,ab OR "induced labor":ti,ab OR "induced labour":ti,ab OR "abdominal delivery":ti,ab OR "Abdominal Deliveries":ti,ab OR Postcesarean*:ti,ab OR dystocia:ti,ab OR Parity:ti,ab OR "Maternal Welfare":ti,ab OR Gravidity:ti,ab OR "spontaneous abortion":ti,ab OR "spontaneous abortions":ti,ab OR "menstrual regulation":ti,ab OR "legal abortion":ti,ab OR "legal abortions":ti,ab OR (Pregnan*:ti,ab,de,tn NOT pregnanolone) OR Childbirth:ti,ab,de,tn OR "child bearing":ti,ab |
| 12 | #1 AND #2 AND #3 |
| 13 | (('Maternal Mortality'/exp OR 'Maternal Death'/exp OR "maternal mortality":ti,ab) AND validation:ti,ab) |
| 14 |  |
| 15 | #12 OR #13 AND [1990-2016]/py |
| 16 | #15 NOT #8 |

#### Russian search terms:

| **#** | **Searches (Web of Science – Russian Index)** |
| --- | --- |
| 1 | («недостаток информации» OR «утерянные данные» OR «дефекты сбора данных» “несообщение” OR «сокрытие» OR «несообщённый» OR «сокрытый» OR «несообщённая» OR «сокрытая» OR «скрытый» OR «скрытая» OR «сокрытые» OR «скрытые» OR «качество информации» OR «качество данных» OR «официальные данные» OR «официальные цифры» OR «официальная статистика» OR «национальная статистика» OR «национальные данные» OR «многочисленные источники» OR «множественные источники» OR «сцепленные данные» OR «связанные данные» OR «незарегистрированные» OR «незарегистрированный» OR «незарегистрированная» OR «не зарегистрированный» OR «не зарегистрированная» OR «не зарегистрированные» OR  «отказ от регистрации» OR «регистрация не проводилась» OR «не регистрировалась» OR «не регистрировался» OR «не регистрировались» OR «причина ошибки» OR «источник ошибки» OR «причины ошибки» OR «источники ошибки» OR «причина ошибок» OR «источник ошибок» OR «причины ошибок» OR «источники ошибок» OR «ошибочная классификация» OR «ошибка классификации» OR «ошибка в классификации» OR «неправильная классификация» OR «неверная классификация» OR «неправильная группировка» OR «неверная группировка» OR «ошибка в группировке» OR «ошибочная группировка»  OR «поздняя материнская смертность» OR «поздней материнской смертности» OR «позднюю материнскую смертность» OR «конфиденциальный запрос» OR «конфиденциальное расследование» OR «закрытая информация» OR «закрытые сведения» OR «закрыть информацию» OR «утаить информацию» OR «утаённая информация» OR «сбор информации» OR «сбора информации» AND («методы» OR «стандарты» OR «механизм» OR «техника» OR «алгоритм» OR «методика» OR «аудит» OR «надзор» OR «популяционный надзор» OR «здоровье населения» OR «состояние здоровья населения» OR «здоровье популяции») |
| 2 | («материнская смертность» OR «акушерская смертность» OR «акушерско-гинекологическая смертность» OR «послеродовая смертность» OR «смерть в родах» OR «родовая смертность» OR «гибель рожениц» OR «гибель родильниц» OR «смертность рожениц» OR «смертность родильниц») |
| 3 | 1 AND 2 |
| 4 | («недостаток информации» OR «утерянные данные» OR «дефекты сбора данных» “несообщение” OR «сокрытие» OR «несообщённый» OR «сокрытый» OR «несообщённая» OR «сокрытая» OR «скрытый» OR «скрытая» OR «сокрытые» OR «скрытые» OR «качество информации» OR «качество данных» OR «официальные данные» OR «официальные цифры» OR «официальная статистика» OR «национальная статистика» OR «национальные данные» OR «многочисленные источники» OR «множественные источники» OR «сцепленные данные» OR «связанные данные» OR «незарегистрированные» OR «незарегистрированный» OR «незарегистрированная» OR «не зарегистрированный» OR «не зарегистрированная» OR «не зарегистрированные» OR  «отказ от регистрации» OR «регистрация не проводилась» OR «не регистрировалась» OR «не регистрировался» OR «не регистрировались» OR «причина ошибки» OR «источник ошибки» OR «причины ошибки» OR «источники ошибки» OR «причина ошибок» OR «источник ошибок» OR «причины ошибок» OR «источники ошибок» OR «ошибочная классификация» OR «ошибка классификации» OR «ошибка в классификации» OR «неправильная классификация» OR «неверная классификация» OR «неправильная группировка» OR «неверная группировка» OR «ошибка в группировке» OR «ошибочная группировка»  OR «поздняя материнская смертность» OR «поздней материнской смертности» OR «позднюю материнскую смертность» OR «конфиденциальный запрос» OR «конфиденциальное расследование» OR «закрытая информация» OR «закрытые сведения» OR «закрыть информацию» OR «утаить информацию» OR «утаённая информация» OR «сбор информации» OR «сбора информации» AND («методы» OR «стандарты» OR «механизм» OR «техника» OR «алгоритм» OR «методика» OR «аудит» OR «надзор» OR «популяционный надзор» OR «здоровье населения» OR «состояние здоровья населения» OR «здоровье популяции») OR «Проверка» OR«отсутствующие данные» OR «пропущенные данные» OR «пропущенные цифры» OR «неточные данные» OR «неточные цифры» OR «скрытые числа» OR «скрытые цифры» OR «скрытые числа» OR «скрытые цифры» OR «скрытые данные» OR «скрытые данные») |
| 5 | («Материнская смертность» OR«Акушерская смертность» OR «Акушерско-гинекологическая смертность» OR «послеродовая смертность» OR «смерть при родах» OR «смертность при рождении» OR «смерть рожениц» OR «смерть рожениц» OR «смертность родильниц» OR «материнская смерть») |
| 6 | #4 AND #5 |
