# Supplementary material for: Incompleteness and misclassification of maternal death recording: a systematic review and meta-analysis
Source: BMC Pregnancy Childbirth. 2023 Nov 15;23:794. doi: 10.1186/s12884-023-06077-4 (PMC10647144; doi:10.1186/s12884-023-06077-4)
Supplement: Supplementary file 2 — Additional file 2: Supplementary information 2. Study risk of bias scoring form [file 12884_2023_6077_MOESM2_ESM.docx]

## Supplementary information 2: Study risk of bias scoring form

| 1/ Sampling frame | Potential maximum mark |
| --- | --- |
| 1.a. Coverage |  |
| National | 1 |
| Subnational | 0 |
| Facility based & skilled birth attendance >95% | 1 |
| Facility based & skilled birth attendance <95% | 0 |
| Population based | 1 |
| 1.b Investigates all deaths to WRA? |  |
| Yes | 1 |
| No | 0 |
| CIMD | 1 |
| **2/ Robustness of COD classification** |  |
| **2.a. Source of COD information** |  |
| Verbal autopsy only | 1 |
| Medical records | 1 |
| Death certificate where we accept CRVS | 2 |
| Death certificate where we don’t accept CRVS | 1 |
| Medical examiner/post-mortem/forensic records | 3 |
| Any combination of more than one source | 3 |
| **2.b Did the study conduct independent expert review of cause of death?** |  |
| Yes | 1 |
| No | 0 |
| **3/percent of records with missing or incomplete cause of death information** |  |
| 10% or less or CIMD | 1 |
| >10% or not reported | 0 |
|  |  |
