# Supplementary material for: Incompleteness and misclassification of maternal death recording: a systematic review and meta-analysis
Source: BMC Pregnancy Childbirth. 2023 Nov 15;23:794. doi: 10.1186/s12884-023-06077-4 (PMC10647144; doi:10.1186/s12884-023-06077-4)
Supplement: Supplementary file 3 — Additional file 3: Supplementary information 3. Study risk of bias score for included studies [file 12884_2023_6077_MOESM3_ESM.docx]

Supplementary information 3 Study risk of bias score for included studies

| **Study** | **Coverage** | **COD assessment** | **Deaths with insufficient information** | **Total** |
| --- | --- | --- | --- | --- |
| Abalos et al, 2019 | 3 | 2 | 0 | 5 |
| Abouchadi et al. 2018 | 2 | 4 | 0 | 6 |
| AIHW, 2020 | 2 | 4 | 0 | 6 |
| Anwar et al, 2018 | 1 | 1 | 0 | 2 |
| Baeva et al, 2018 | 1 | 2 | 0 | 3 |
| Boutin et al.2020 | 1 | 2 | 0 | 3 |
| Boyd et al, 2017 | 0 | 2 | 0 | 2 |
| Catalano et al. 2020 | 1 | 1 | 1 | 3 |
| Constantén et al. 2018 | 3 | 4 | 1 | 8 |
| Davis et al, 2017 | 2 | 0 | 0 | 2 |
| Deneux-Tharaux, 2005 | 3 | 4 | 1 | 8 |
| Donati et al, 2018 | 2 | 2 | 0 | 4 |
| Garces et al, 2012 | 2 | 4 | 0 | 6 |
| Horon, 2005 | 1 | 4 | 1 | 6 |
| Kodan et al, 2017 | 3 | 3 | 0 | 6 |
| Kodio et al, 2002 | 2 | 4 | 0 | 6 |
| Laura et al, 2020 | 2 | 3 | 0 | 5 |
| Lin et al, 2019 | 2 | 2 | 1 | 5 |
| Lomia et al., 2018 | 3 | 4 | 1 | 8 |
| MBRACE-UK, 2021 | 3 | 4 | 1 | 8 |
| Mswia et al, 2003 | 2 | 3 | 0 | 5 |
| Mwaniki, Edwards & Kizito, 2020 | 0 | 2 | 0 | 2 |
| O'hare et al, 2020 | 3 | 4 | 1 | 8 |
| Qomariyah et al. 2020 | 2 | 3 | 1 | 6 |
| Sesmero et al, 2016 | 1 | 3 | 0 | 4 |
| Songane & Bergström, 2002 | 2 | 4 | 0 | 6 |
| Wu et al, 2015 | 3 | 2 | 0 | 5 |
| Vangen et al., 2017 | 3 | 4 | 0 | 7 |
| Zakariah et al, 2009 | 2 | 4 | 0 | 6 |
